# Efficient Generation of Paired Single‐Cell Multiomics Profiles by Deep Learning

**DOI:** 10.1002/advs.202301169

**Published:** 2023-04-28

**Authors:** Meng Lan, Shixiong Zhang, Lin Gao

**Affiliations:** ^1^ School of Computer Science and Technology Xidian University Xi'an Shaanxi 710071 China

**Keywords:** deep learning, multiomics, single cells

## Abstract

Recent advances in single‐cell sequencing technology have made it possible to measure multiple paired omics simultaneously in a single cell such as cellular indexing of transcriptomes and epitopes by sequencing (CITE‐seq) and single‐nucleus chromatin accessibility and mRNA expression sequencing (SNARE‐seq). However, the widespread application of these single‐cell multiomics profiling technologies has been limited by their experimental complexity, noise in nature, and high cost. In addition, single‐omics sequencing technologies have generated tremendous and high‐quality single‐cell datasets but have yet to be fully utilized. Here, single‐cell multiomics generation (scMOG), a deep learning‐based framework to generate single‐cell assay for transposase‐accessible chromatin (ATAC) data in silico is developed from experimentally available single‐cell RNA‐seq measurements and vice versa. The results demonstrate that scMOG can accurately perform cross‐omics generation between RNA and ATAC, and generate paired multiomics data with biological meanings when one omics is experimentally unavailable and out of training datasets. The generated ATAC, either alone or in combination with measured RNA, exhibits equivalent or superior performance to that of the experimentally measured counterparts throughout multiple downstream analyses. scMOG is also applied to human lymphoma data, which proves to be more effective in identifying tumor samples than the experimentally measured ATAC data. Finally, the performance of scMOG is investigated in other omics such as proteomics and it still shows robust performance on surface protein generation.

## Graphical abstract

scMOG can accurately perform cross‐omics generation in silico between RNA and ATAC and generate paired multiomics data with biological meanings when one omics is experimentally unavailable. The generated ATAC exhibits comparable performance to that of the experimentally measured counterparts, making it more effective in identifying tumor samples in human lymphoma data. Additionally, scMOG shows robust performance on surface protein generation.

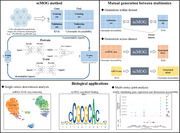

## 1 Introduction

Advances in single‐cell isolation and barcoding have allowed measurements of gene expression, chromatin accessibility, methylation, and protein abundance at single‐cell resolution learning to unprecedented biological insights compared to the bulk sequencing era.^[^ ^1^, ^2^ ^]^ For example, scRNA‐seq (single‐cell RNA sequencing) characterizes the transcriptional state of individual cells and reveals cell subpopulations, while scATAC‐seq (single‐cell Assay for Transposase‐Accessible Chromatin using sequencing) reveals chromatin heterogeneity. This, in turn, enables us to gain a deeper understanding of biological processes such as immune heterogeneity and tumor dynamics at a nuanced level.^[^ ^3^, ^4^, ^5^, ^6^ ^]^ However, these techniques only characterize one layer of single cells preventing us from comprehensively understanding cellular processes.

This gap promotes the development of single‐cell multiomics sequencing protocols that can simultaneously profile multiple molecular modalities from the same single cell. For instance, REAP‐seq^[^ ^7^ ^]^ and CITE‐seq^[^ ^8^ ^]^ measure transcriptome and protein simultaneously, while SHARE‐seq^[^ ^9^ ^]^ and SNARE‐seq^[^ ^10^ ^]^ measure transcriptome and chromatin accessibility simultaneously. These paired omics measurements provide a comprehensive understanding of how different cellular mechanisms interact with each other. For example, Jia et al.^[^ ^11^ ^]^ studied cardiac progenitor cells in mice by analyzing intercellular transcriptome and chromatin accessibility heterogeneity, which allowed the identification of previous unknown cardiac subpopulations; Sheng et al.^[^ ^12^ ^]^ discovered some key loci for the disease by studying methylation and gene expression changes in diabetic kidney disease. Multiomics measurements can provide more knowledge than single omics and lead to insightful inferences that cannot be obtained from any single omics approach.^[^ ^13^ ^]^

However, paired measurements profiled by single‐cell multiomics protocols are inherently more complex and exhibit greater noise compared to single omics data. A crucial factor contributing to it is the technical complexity involved in such protocols: i) clinical specimens used for measuring multiple molecules in the same cell are often flash‐frozen or embedded in paraffin, which can disrupt the cytoplasmic membrane and lead to erroneous conclusions;^[^ ^14^ ^]^ ii) prolonged exposure to dissociative enzymes or mechanical dissociation during the preparation of fresh tissues can cause the degradation of mRNA and proteins, thereby reducing the quality and quantity of the data.^[^ ^15^ ^]^ Furthermore, the high costs associated with single‐cell multiomics profiling protocols pose a significant barrier to their widespread application.

According to the central dogma of molecular biology, genetic information flows only in one direction, from DNA to RNA, to protein, or RNA directly to protein. This concept may inspire us to generate unmeasured omics data from measured single‐cell omics data in the same tissue. For instance, BABEL^[^ ^16^ ^]^ enables cross‐modality translation between multiomics profiles but does not perform well in translating from transcriptome to chromatin accessibility. cTP‐net^[^ ^17^ ^]^ is specifically developed to impute surface proteins from the transcriptome. Additionally, some multi‐omics joint analysis methods, such as scMM^[^ ^18^ ^]^ and Multivi,^[^ ^19^ ^]^ also enable cross‐omics generation, but their generation performance is unsatisfactory as they are initially designed for data integration. Furthermore, these methods tend to perform poorly when generating across datasets.

In this study, we present a deep learning framework, termed scMOG, that allows cross‐omics generation, enabling to efficient generate paired measurements of single‐cell multiomics. scMOG utilizes pre‐training to jump out of local optimum and employs different loss functions tailored to each specific omics data type (e.g., scRNA‐seq counts ≈10k mRNA and scATAC‐seq counts ≈100k peak states). We assess the cross‐omics generation performance of paired multiomics profiles when only one single‐cell omics measurement is available experimentally, but outside of the training datasets. In addition, we investigate the biological significance of the generated profiles both individually and in conjunction with the experimental measurements in a comprehensive manner. Finally, we explore the performance of scMOG on other omics data, such as single‐cell proteome.

## 2 Results

### 2.1 Method Overview

**Figure 1:**
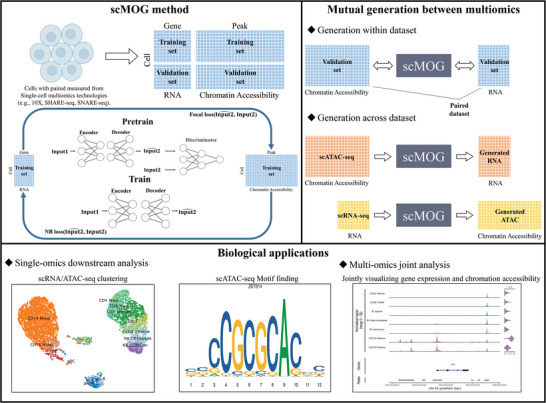
Overview of the proposed scMOG. scMOG uses paired multiomics sequencing dataset (SHARE‐seq or SNARE‐seq) as input and learns the relationships between different omics. The workflow of the scMOG model is as follows. The paired dataset is divided into a training set and a validation set. When generating ATAC from RNA, we use RNA data as Input1 and ATAC data as Input2, and vice versa. In the pre‐training phase, scMOG is based on an autoencoder (AE) and a discriminator that is connected to the AE for pre‐training to obtain the initial parameters. In the training phase, scMOG removes the discriminator and trains the model in two‐generation tasks of ATAC to RNA and RNA to ATAC separately. We evaluate the performance of scMOG in two scenarios. First, we evaluate the cross‐omics generation on the validation set (i.e., within datasets). Second, we leverage the trained scMOG to explore generative performance on single‐omics data (scRNA‐seq or scATAC‐seq dataset) from the same tissue with the training paired dataset (i.e., across datasets). The output of scMOG can be applied for single‐omics downstream analysis and multi‐omics joint analysis.

scMOG uses a deep generative model to perform cross‐omics generation at single‐cell resolution (**Figure** **1**). Specifically, scMOG takes paired single‐cell multimodal data as input, for instance, RNA and ATAC data sequenced from the same single cells. To achieve accurate cross‐omics generation, scMOG adopts a two‐step approach, as illustrated in Figure 1.

1) Pre‐training (e.g., RNA is used to generate ATAC): This step has shown to outperform in handling highly sparse data, and it also improves the robustness of scMOG.^[^ ^20^ ^]^ First, the RNA encoder and the ATAC decoder are constructed. The encoder networks map RNA data into a low‐dimensional latent space, and the latent representation is mapped back to the decoder to generate paired ATAC data. Second, the generated ATAC data is input into the discriminator with the experimentally measured ATAC data, enabling adversarial learning to initialize the model parameters. This pre‐training enables scMOG to optimize quickly and jump out of the local optima in subsequent training compared to starting from scratch.
2) Training: The discriminator is removed and the encoder and decoder remain. We use negative binomial (NB) distribution loss to quantify the difference between generated RNA data and scRNA‐seq measurements. Given that the ATAC data exhibit higher dimensionality and sparsity than RNA data, we introduce Focal loss^[^ ^21^ ^]^ for ATAC data generation in the model training. Focal loss is more suitable for handling extremely sparse and unbalanced binary data (e.g., scATAC‐seq) than the binary cross‐entropy loss, and it can improve the model's generation capability.

### 2.2 scMOG Performs High‐Accuracy Cross‐Omics Generation across Datasets

The datasets used in this section are paired datasets which are summarized in Table S1, Supporting Information. These paired datasets were collected from multiple cell lines including peripheral blood mononuclear cells (PBMC), colon adenocarcinoma COLO‐320DM (DM) and COLO‐320HSR (HSR) cells, lymphoblastoid GM12878 cells, Flash‐Frozen Lymph Node with B Cell Lymphoma, and adult mouse brain cerebral cortex and brain, from two species (human and mouse). All the paired datasets jointly profiling transcriptome and chromatin accessibility were generated using 10x Genomics’ multiomics platform, SHARE‐seq, and SNARE‐seq.

The performance of scMOG's cross‐omics generation is evaluated on above paired datasets that are split into training, testing, and validation datasets (i.e., “within datasets”). Details of the data splits can be found in the Experimental Section. In addition, scMOG is trained on human PBMC (11 909 cells) and GSE160148 (DM and HSR; 24 508 cells) datasets, and then leverage the trained scMOG on other paired PBMC datasets to explore its generative performance on datasets out of the training ones (i.e., “across datasets”).

**Figure 2:**
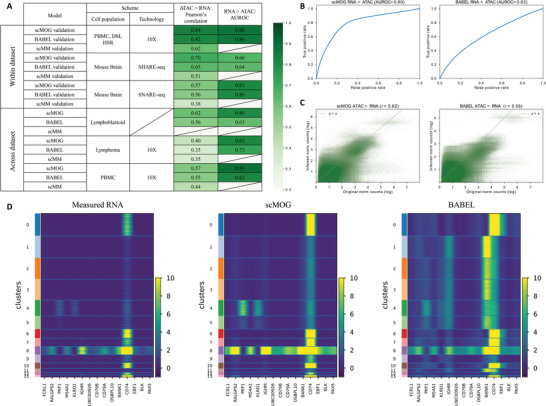
Benchmark evaluation on multiple datasets. A) Performance evaluation of scMOG in two scenarios: within dataset and across dataset. The goal of this evaluation is to measure the ability of scMOG to generate ATAC and RNA data and compare it with the performance of two benchmark methods, BABEL and scMM. B) Performance of RNA to ATAC generation on GM12878 data is evaluated using AUROC, and C) the performance of ATAC to RNA generation on the same dataset is evaluated using Pearson's correlation. In the density scatterplot, the *x*‐axis represents measured RNA and the *y*–axis represents generated RNA. D) Comparison of the RNA data generated from the scMOG and BABEL methods with the measured RNA data. Both methods are trained on single‐cell human PBMC (11 909 cells) and GSE160148 (24 508 cells) and tested on another single‐cell Human PBMC data (10 691 cells). The heatmaps consist of 15 highly variable genes' expression patterns and the hierarchically clustered cells. Each column in the heatmap corresponds to a specific variable gene, while each row corresponds to a particular cell. The colors range from yellow to green, indicating high to middle expression, while green to black colors indicate low to null expression.

To estimate its performance, we consider two benchmark methods, BABEL^[^ ^16^ ^]^ and scMM.^[^ ^18^ ^]^ We compute Pearson's correlation and the area under the receiver operating characteristic (AUROC) separately for the cross‐omics generation performance of ATAC to RNA and RNA to ATAC, as the continuous RNA expression data and binary ATAC data are different in nature. For the “within datasets” scenario, both scMOG and BABEL perform significantly better than scMM, while scMOG slightly outperforms BABEL (**Figure** **2**A and Figure S1, Supporting Information).

In the “across datasets” scenario, scMOG shows a greater advantage over benchmark methods, particularly in generating ATAC data from RNA data. For example, we trained scMOG on human PBMC (11 909 cells) and GSE160148 (24 508 cells) datasets and tested it on the GM12878 dataset (3509 cells). In this case, scMOG achieved a Pearson's correlation of 0.62 when generating RNA from ATAC, while BABEL only achieve 0.56. Similarly, scMOG achieved an AUROC of 0.80 when generating ATAC from RNA, while BABEL only achieved 0.63 (Figure 2B,C). Additionally, scMOG maintained an AUROC (RNA to ATAC) of over 0.8 on all test datasets, and its generation performance remained stable during cross‐validation with random division (Table S2, Supporting Information). Moreover, we demonstrate the unique advantage of scMOG over BABEL by presenting an example of both methods on RNA generation in the “across datasets” scenario. Both methods were trained on Human PBMC (11 909 cells) and GSE160148 (24 508 cells) datasets and tested on another Human PBMC data (10 691 cells). Figure 2D displays the experimentally measured RNA profiles (10 691 cells) and the generated RNA profiles by scMOG and BABEL from experimentally measured ATAC data. The heatmaps in Figure 2D depict the expression patterns of 15 highly variable genes and the hierarchical clustering of cells. In Figure 2D, BABEL exhibits significant RNA expression loss and misexpression in this “across datasets” scenario, whereas the RNA profiles generated by the proposed scMOG can recover most of the gene expression patterns consistent with the measured RNA data.

To explore the effect of the number of peak calling features used on RNA generation, we randomly downsampled the ATAC features from their original peak calling dimension to four lower dimensions on the SHARE‐seq mouse brain dataset, which consisted of 3293 cells and 200 255 peaks after pre‐processing. We then examined the Pearson's correlation coefficient between measured RNA and generated RNA using different numbers of peak calling features from ATAC data and the results are tabulated in Table S3, Supporting Information. We found that scMOG exhibited a slight performance degradation when the number of peaks was dropped from 200 255 to 10 000, and decreased significantly at 5000 peaks. This indicates that although RNA generation performance improves with an increase in the number of peak calling features, it is not improved to a large extent, mainly due to the inherent sparsity of scATAC‐seq data.

Moreover, we investigate the effectiveness of the pre‐training step in scMOG. We apply the same pre‐processing to both scMOG and the No_pretrain model (which is scMOG without pre‐training) and evaluate their performance on the aforementioned datasets (Table S4, Supporting Information). Our results indicate that the pre‐training step improves the cross‐omics generation ability, particularly in the case of across datasets.

### 2.3 scMOG Can Generate Biologically Meaningful Omics Data

Our main concern is whether the generated data (RNA or ATAC) is biologically meaningful and can be used for downstream single‐cell analysis in the same manner as the sequenced (scRNA‐seq or scATAC‐seq) data. In this section, we aim to explore biological insights from the generated RNA or ATAC data. scMOG is trained on two paired transcriptome and chromatin accessibility datasets, namely human PBMC (11 909 cells) and GSE160148 (24 508 cells). The testing datasets used in this section are unpaired datasets (i.e., only have one omics data) listed in Table S1, Supporting Information. BABEL is used as the benchmark for comparison.

**Figure 3:**
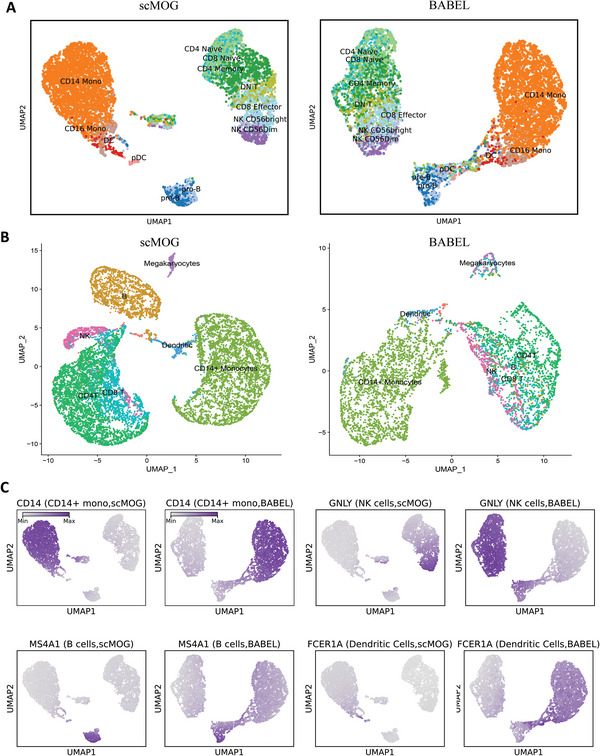
scMOG can generate biologically meaningful omics data on unpaired PBMCs. A) UMAP visualization of scMOG's (left) and BABEL's (right) RNA expression profiles generated from scATAC‐seq data (8633 cells), which are colored by ATAC‐derived cell type identities. B) UMAP visualization of scMOG's (left) and BABEL's (right) ATAC data generated from scRNA‐seq data (11 769 cells), which are colored by RNA‐derived cell type identities. C) Feature plots for CD14 (a marker for CD14+ monocytes), GNLY (a marker for NK cells), MS4A1(a marker for B cells), and FCER1A (a marker for DC cells). The scatter plot represents a UMAP representation of the generated RNA expression. We colored each cell according to the gene expression generated by scMOG and BABEL.

First, we apply the trained scMOG to the scATAC‐seq human PBMC data (8633 cells) to generate hypothetical paired RNA expression profiles. After performing data normalization and log transformation on the generated RNA expression data, all cells are visualized using the uniform manifold approximation and projection (UMAP) algorithm.^[^ ^22^, ^23^ ^]^ Each cell is colored with the cell labels transferred from real measurements of ATAC data (Figure S2, Supporting Information). As depicted in Figure S2, Supporting Information, the scATAC‐seq human PBMC data contains four main cell clusters:^[^ ^16^ ^]^ CD4, CD8, and natural killer (NK) cells, B cells, CD14+/CD16+ monocytes and dendritic cells (DCs), and pDC cells. As shown in **Figure** **3**A, the generated RNA measurements by scMOG can well delineate the four major cell populations, while BABEL can only yield two large populations. This suggests that scMOG can identify the complex relationships between different omics and leverage them to generate biologically meaningful and denoised RNA expression data.

In addition, we also investigate the performance of imputing marker gene expression for specific cell types (Figure 3C and Figure S3, Supporting Information). CD14 is a marker for CD14+ monocytes. By coloring the CD14 expression against each cell in UMAP plots, we find that both scMOG and BABEL generated CD14 expression consistent with CD14+ monocytes, with almost perfect overlap between the two (Figure 3C). For GNLY (a marker for NK cells) and MS4A1 (a marker for B cells), scMOG accurately matches GNLY to NK cells and MS4A1 to B cells, whereas BABEL does not perform well (Figure 3C). We also examined FCER1A (a marker for DC cells), IL7R (a marker for CD4 T cells), LYZ (a marker for CD14 monocytes), NKG7 (a marker for NK cells), CD8A (a marker for CD8 T cells), and CST3 (a marker for DC cells) (Figure 3C and Figure S3, Supporting Information). It can be observed that scMOG can accurately impute the gene expression of each marker gene to match the correct cell types. In contrast, BABEL can impute marker genes corresponding to large clusters but performs poorly for DC cells, B cells, and NK cells which are clusters with small cell numbers. These finds suggest that scMOG can make specific inferences for individual cells, and the generated gene expression can be used in downstream analysis.

Moreover, we also tested the trained scMOG on another scRNA‐seq human PBMC data (11 769 cells) to generate its hypothetical paired ATAC profiles. We preprocess the generated ATAC data to remove low‐quality cells (details of the quality control process are provided in the Experimental Section). Out of the 11 522 cells generated by scMOG, 11 510 cells passed the quality control filter. In contrast, BABEL only retained 4930 cells after filtering out the majority of the B cells. This indicates that the majority of the ATAC data generated by scMOG is of high quality. We visualized the ATAC data generated by scMOG and BABEL with and without the quality control process separately in UMAP plots and colored each cell using cell labels transferred from the measured RNA expressions (Figure 3B and Figure S4, Supporting Information). We observed that scMOG precisely delineates the individual cell clusters both with and without quality control. However, BABEL was unable to obtain clear clustering visualization results without quality control. In addition to the datasets mentioned above, we also tested scMOG on additional PBMC data (4623 cells) and mouse brain nuclei datasets. Figure S5A, Supporting Information illustrates the stable generation performance across datasets in PBMC tissue. For the mouse brain nuclei datasets, scMOG and BABEL were trained on a paired multiomics dataset (10X Single Cell Multiome ATAC + Gene Expression; 23 990 cells) and then tested on a scRNA‐seq mouse brain nuclei dataset (7377 cells) to generate ATAC data. Figure S5B, Supporting Information illustrates that scMOG can effectively distinguish different cell types, while BABEL performs poorly.

### 2.4 Generated Chromatin Accessibility Profiles Perform Well in Joint Single‐Cell Multiomics Downstream Analysis

**Figure 4:**
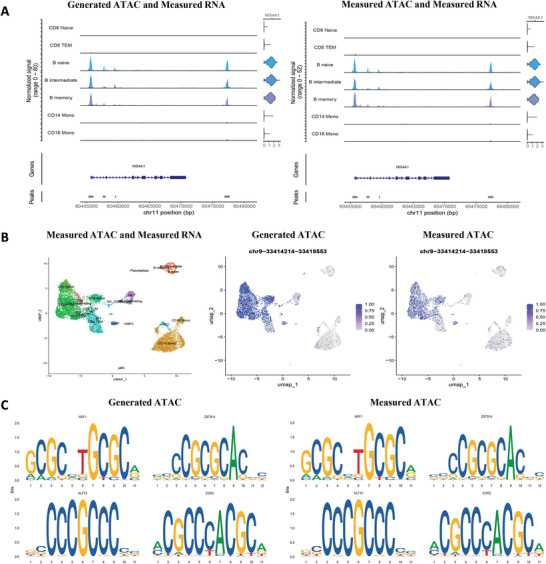
Generated chromatin accessibility profiles exhibit good performance in downstream joint single‐cell multiomics analysis on paired human PBMC data (10 691 cells). A) Coverage plots for clusters within MS4A1 regions (a marker for B cells) for the generated ATAC and measured RNA (left) and the measured ATAC and measured RNA (right). B) Using UMAP plots from the joint analysis of measured RNA and measured ATAC (left), we identify differentially accessible regions between CD4 naive and CD14 mono for generated ATAC (middle) and measured ATAC (right). C) Motif plots for representative motifs between CD4 naive versus CD14 mono from generated ATAC (left) and measured ATAC (right).

In this section, we investigate the performance of joint multiomics analysis using experimental measurements, such as scRNA‐seq data, and the corresponding hypothetical paired chromatin accessibility profiles generated by scMOG. Similar to the previous section, scMOG is trained on two paired datasets: human PBMC (11 909 cells) and GSE160148 (24 508 cells). A paired human PBMC data (10 691 cells) included in Table S1, Supporting Information is used as the testing data. We leverage the trained scMOG to generate ATAC data from the measured RNA expression data of testing human PBMC data for the joint multiomics downstream analysis. The benchmark for the downstream analysis is experimentally measured paired multiomics profiles (i.e., the testing PBMC). For the joint multiomics downstream analysis, we analyze the coverage plots of the MS4A1, CD8A, LYZ, and CD14 gene regions (**Figure** **4**A and Figure S6, Supporting Information), which are essential markers for B cells, CD8 T cells, and CD14 monocytes, respectively. The signals from all cells within a cluster are averaged together to visualize the chromatin accessibility of a region and combined with transcriptome information. This makes it easy to compare the chromatin accessibility in a given region for different cell types and overlay gene expression information for different genes. The coverage plots demonstrate that the scMOG‐generated chromatin accessibility can reconstruct the peaks specifically detected in cell subsets.

Furthermore, we perform differential accessibility tests to detect differentially accessible regions or peaks across various cell types. To benchmark our results, we present the clustering of experimentally measured multi‐omics data in Figure 4B. Next, we contrast the differential accessibility peaks between CD4 naive cells and CD14 monocytes (Figure 4B). The genomic region Chr9‐33414214‐33418553 should exhibit high expression in the CD4 naive cell region but not in the CD14 monocyte. The specific accessible peak generated by scMOG elevates the expression in CD4 naive cells while reducing it in CD14 monocytes, thus accurately reflecting the biological phenomenon. This indicated that scMOG is capable of generating denoised chromatin accessibility data. In addition, we investigate differentially accessible regions between CD14 monocytes and NK cells, CD14 monocytes and B naïve cells, and NK and B naïve cells, among others, separately (Figure S7, Supporting Information). These finds further support scMOG's ability to generate paired omics data.

ATAC‐seq studies regions of chromatin that are open and contain a significant amount of motif information related to transcription initiation.^[^ ^24^ ^]^ This motif information can be used to identify regulatory transcription factors (TFs) in a database, which can inform downstream experiments. In our study, we searched for overrepresented DNA sequence motifs in the set of differentially accessible peaks between various cell types, including CD4 naive cells and CD14 monocytes, NK and B naive, CD14 mono and B naive, CD4 naive and CD4 TEM, using the ATAC data generated by scMOG. Seqlogo plots (Figure 4C and Figure S8, Supporting Information) illustrate the results, which are consistent with the findings obtained from measured ATAC data. These findings suggest that scMOG has a high potential for generating chromatin accessibility data that can be used for single‐cell multiomics integration analysis, with the aim of identifying TFs implicated in regulating these cells.

### 2.5 scMOG Can Be Applied to Disease Samples

**Figure 5:**
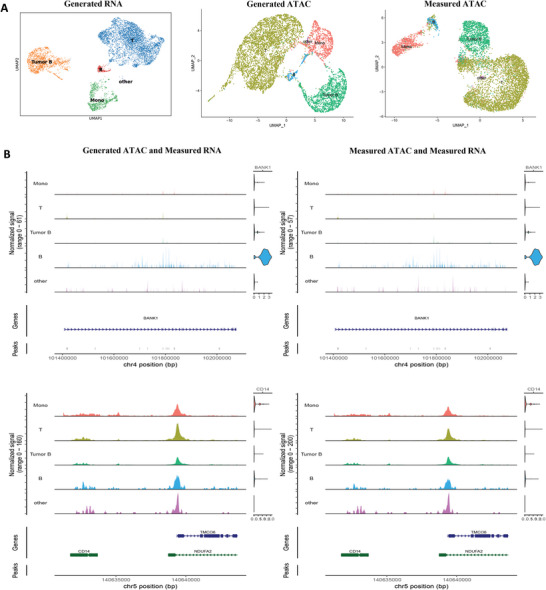
scMOG demonstrates accurate generation performance on lymph node tumor data (14 566 cells). A) UMAP plots display the measured RNA (left), generated ATAC (middle), and measured ATAC (right). Each cell is colored according to the measured RNA expression. The UMAP plot of RNA expression serves as a benchmark to assess its retention in the ATAC expression. B) Coverage plots of clusters within regions of BANK1 (an attenuator of BCR activation pathway that is repressed in tumor cells relative to normal B cells) and CD14 (a marker for CD14+ monocytes) for the generated ATAC and measured RNA (left), and the measured ATAC and measured RNA (right) are shown.

To further explore the potential of scMOG in complex disease samples, we applied it to a dataset of tumor B cells. These samples were obtained from flash‐frozen intra‐abdominal lymph node tumors from a patient with diffuse small lymphocytic lymphoma. The dataset contains a total of 14 566 cells and measures chromatin accessibility and RNA expression simultaneously. We used 50% of the dataset as a training set and the other 50% as a testing set. We compared the generated ATAC from scMOG with the real measured ATAC data and found the AUROC to be 0.80 (Figure S9, Supporting Information). We use the measured RNA expression‐derived cell type identities to color the scMOG‐generated ATAC data for UMAP clustering (**Figure** **5**A). The UMAP plots demonstrated that the generated ATAC data accurately identified tumor B cells as well as healthy cell types such as T cells, monocytes, and B cells, consistent with the clustering of ATAC data obtained through experimental measurement.

Additionally, scMOG allowed for a more nuanced analysis of the disease. BANK1 acts as an important adaptor molecular to link B‐cell receptor (BCR)‐mediated signaling to the generation of intracellular secondary messengers in B cells.^[^ ^25^ ^]^ With the ATAC data generated by scMOG, we analyzed the coverage plots of disease‐related genes such as BANK1 (Figure 5B). We found that BANK1 was repressed in tumor B cells relative to normal B cells, consistent with the measured ATAC data. Our results suggest that BANK1 has a negative effect on cell proliferation and BANK1 inactivation may contribute to lymphoma by promoting cell proliferation, as demonstrated in.^[^ ^26^ ^]^ We also analyzed the coverage plots of healthy cell marker genes such as CD14, MS4A1, and IL7R (Figure 5B and Figure S10, Supporting Information), and the results suggest that the generated paired multiomics profiles can nearly capture the whole biological information when compared with the experimentally measured multiomics profiles.

We investigated whether the ATAC data generated by scMOG could capture the heterogeneity among individual patients. To achieve this, we applied scMOG to a multi‐omics dataset of Alzheimer's disease (AD) mouse brains consisting of 33 459 cells (66 914 peaks and 32 286 genes) from 12 transgenic (Tg) and wild‐type (WT) mice. A previous study has reported that the gene Slc1a3 is one of the high‐affinity glutamate transporters that mediate the cellular uptake of glutamate and that its dysfunction can lead to the pathogenesis of AD.^[^ ^27^ ^]^ Using the ATAC data generated by scMOG, we analyzed the coverage plots of Slc1a3 in four 2.5‐month‐old transgenic (Tg 2.5 mo‐1 and Tg 2.5 mo‐2) and wild‐type (WT 2.5 mo‐1 and WT 2.5 mo‐2) mice (Figure S11, Supporting Information). Our analysis revealed that SLc1a3 exhibits heterogeneity among individuals and presents large differences between Tg and WT mice, which is consistent with the measured ATAC data.

### 2.6 scMOG Can Be Extended to Proteomics

**Figure 6:**
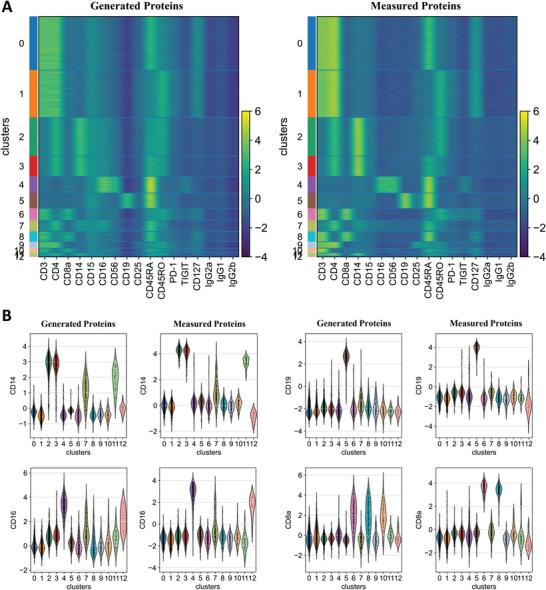
Extending scMOG to generate single‐cell protein abundances on human PBMC data (5527 cells). A) Heatmaps were constructed from generated (left) and measured (right) surface protein counts. Rows and columns represent 17 shared surface protein markers and 13 clusters discovered by measured protein, respectively. B) Violin plots were used to visualize the feature values of CD14 protein, CD19 protein, CD16 protein, and CD8a protein. We examined the generated expression (left) and measured expression (right).

Cell surface proteins can serve as primary targets for therapeutic interventions and markers of specific cellular functions.^[^ ^28^, ^29^, ^30^ ^]^ In this study, we sought to apply scMOG to proteomics to generate protein data from the transcriptome (see Experimental Section for details on model architecture). We applied the scMOG on two healthy donors’ PBMC datasets that jointly measured RNA and 17 protein epitopes, using 7865 cells as the training set and 5527 cells as the testing set (Table S5, Supporting Information). The generated cell surface protein data from the transcriptome achieved a Pearson's correlation of 0.81 with its real experimental expressions (Figure S12, Supporting Information). For the 13 clusters found by true measurement of protein clustering (Figure S13A, Supporting Information), we visualized the expression levels of 17 surface proteins for both generated and experimentally measured by using heatmaps (**Figure** **6**A). The results show that the surface protein data generated by scMOG captures the features of the experimentally measured data well.

Cell surface proteins exhibit larger variation among cell types and higher consistency within cell types than their respective genes' RNA expression, which differs significantly from both.^[^ ^17^ ^]^ As a result, protein levels act as interpretable intermediate features for detecting and labeling cell states that characterize cell types more precisely than RNA levels of corresponding marker genes. For instance, CD14 is a marker for CD14 monocytes, CD19 is a marker for B cells, CD16 is a marker for CD16 monocytes and NK cells, and CD4 and CD8 can distinguish CD4+ T cells from CD8+ T cells. In addition, CD45RA is a naive antigen and CD45RO is a memory antigen; naive T cells can be further separated into memory T cells by the abundance of CD45RA/CD45RO. We used violin plots to illustrate the distribution of protein abundances among cells for both measured protein and generated protein counts (Figure 6B and Figure S13B–D, Supporting Information). The results show that the surface protein data generated by scMOG can accurately distinguish the different cell types, consistent with the results of measured surface protein data. Our findings demonstrate that scMOG can be flexibly extended to cell surface protein generation when only scRNA‐seq data is available.

## 3 Discussion

In this study, we present a deep learning framework, termed scMOG, which enables the generation of paired single‐cell multiomics profiles in silico, even when only one omics data is experimentally available. scMOG is based on a pretrain‐train paradigm, allowing the model to learn cell‐specific information efficiently and generate missing omics data accurately and robustly. We demonstrate that scMOG performs well in a variety of contexts and is particularly adept at cross‐dataset generation, while preserving biological information. Furthermore, we demonstrated the utility of scMOG in joint multiomics analysis by computationally generating missing data modality. Specifically, we applied scMOG to lymph node tumor data and AD mouse brain data. scMOG exhibited robust performance in these complex disease samples, and furthermore, it effectively captures the heterogeneity among individual patients. Moreover, in addition to the mutual generation of gene expression and chromatin accessibility, we show that scMOG can be extended to generate surface protein abundances.

Despite its strengths, scMOG has certain limitations. Notably, scMOG performed well in cross‐omics generation within the same tissue, but not across different tissue, owing to significant differences in the cell composition of different tissues.^[^ ^31^ ^]^ In the future, we plan to extend scMOG to include more omics data types. As more single‐cell multi‐omics datasets become available, scMOG can be retrained to generate omics data for cross‐tissue analysis. We anticipate that scMOG will have a broad impact as a method for exploring complex regulatory mechanisms of intracellular heterogeneity and analyzing clinical disease samples such as cancer.

## 4 Experimental Section

### Datasets and Pre‐Processing

Table S1, Supporting Information provides a summary of the datasets utilized in this study. To preprocess the scRNA‐seq data, cells with fewer than 200 genes or more than 7000 genes (2500 for mouse data, as described in the original SNARE‐seq paper^[^ ^11^ ^]^) were filtered out and genes located on the sex chromosomes were removed. The data were size‐normalized such that the count values for each cell sum up to the median count per cell. After this, a log transformation was applied and the data were standardized to have a zero mean and unit variance. For the scATAC‐seq data, peaks that occurred in fewer than five cells or more than 10% of cells were filtered out to focus the model on capturing crucial changes among cells. All the preprocessing steps described above were implemented through the Python package Scanpy.^[^ ^32^ ^]^

To partition the training, validation, and test sets, the following approach was adopted. The preprocessed RNA data were clustered using Leiden's algorithm.^[^ ^33^ ^]^ Two larger clusters were designed as the validation and test sets, while the remaining cells form the training set. Compared with a random partitioning method, this approach reduced the degree of similarity among different subsets, and the evaluation metrics on the test set better reflect the model's generalization ability.

### scMOG Model Architecture

The scMOG architecture comprised an RNA encoder, an ATAC decoder, and an ATAC discriminator, with the objective of generating ATAC data from RNA. The RNA encoder mapped the RNA data into a low‐dimensional latent space, and the decoder used this representation to generate the corresponding ATAC data. The discriminator was then used for generative adversarial learning to pre‐train the model. After pre‐training, the discriminator was removed, and the encoder and decoder were subjected to ATAC‐specific training.

The RNA encoder had an input layer dimension equal to the number of genes, and two hidden layers with dimensions of 256 and 64, respectively, before outputting the final 16D learned representation of each cell. The use of multiple layers with decreasing dimensions allowed the network to learn hierarchical representations of the input data. The ATAC decoder mapped the 16D latent representation to a 64D hidden layer, then to a 512D hidden layer, before outputting the peak values. The LeakyRelu function was used in each layer, except the last layer of the decoder, to increase the network's nonlinearity and learn complex biological information. The Sigmoid function was used in the final layer to obtain the probability value of the peak between 0 and 1. During generation, the output was binarized using a threshold of 0.1 to obtain the peaks for each cell. This thresholding step produced binary data that can be used to identify the presence or absence of the peaks in each cell.

In contrast to the previously described model, the scMOG model for generating RNA from ATAC consisted of an ATAC encoder, RNA decoder, and RNA discriminator. The ATAC encoder was structured in reverse order to the ATAC decoder, and the RNA decoder was structured in reverse order to the RNA encoder. Specifically, the ATAC encoder had an input layer with a dimension equal to the number of peaks in the input data. In the scMOG model, the ATAC encoder had two hidden layers of 512 and 64, respectively, before outputting the 16D final learned representation of each cell. The main difference lies in the RNA decoder, which mapped the 256D hidden layer into two outputs, mean and dispersion, using the exponential and softplus activation functions, respectively. Together, these two outputs described the likelihood of observed expression of each gene under a negative binomial distribution.

The RNA discriminator and the ATAC discriminator had the same architecture, which was a five‐layer neural network. The measured and generated values served as input and were then mapped to 1024 dimensions, 256 dimensions, 64 dimensions, 16 dimensions, and 1 dimension. The LeakyRelu function was used between each layer except the last layer, and the last layer did not have an activation function. Xavier_uniform() was used for parameter initialization for each layer in the autoencoders and discriminators. This function initialized the weights randomly, following a uniform distribution with a specific range, in such a way that the mean and variance of the input and output of each layer were approximately equal. In addition, the bias of the last layer of the ATAC decoder was initialized to −2, which was commonly used for 0–1 imbalance to improve the performance of the model. By setting the bias to a negative value, it can help the decoder to quickly learn the sparsity of the data more quickly, which can lead to faster convergence and better performance of the scMOG.

### Optimization Process

During the pre‐training phase, a method similar to the generative model WGAN was adopted.^[^ ^34^ ^]^ The Wasserstein distance was utilized to measure the distance between the measured distribution and the generated distribution, with the aim of minimizing this distance. The Wasserstein distance is defined as follows.

(1) $$W\;\left(P_{1},P_{2}\right)=\begin{array}{c} \inf\\ \gamma \in \prod \left(P_{1},P_{2}\right) \end{array}E_{\left(x,y\right)∼\gamma }\left[∥x-y∥\right]$$

where *P*_1_ denotes the distribution of measured data, while *P*_2_ denotes the distribution of generated data. Together, they constituted the joint probability distribution $\prod (P_{1},P_{2})$. The variable γ satisfied the joint probability distribution, and (x,y) was a sample from γ. The expectations $E_{(x,y)∼\gamma }\left[∥x-y∥\right]$ for different joint distributions γ varied, and the infimum in these expectations defined the Wasserstein distance between the distributions *P*_1_ and *P*_2_.

During the training phase, the ATAC decoder needs to generate values between [0, 1] for each peak. Currently, the binary cross‐entropy is a widely adopted deep learning method for this task, given by,

(2) $$\mathrm{CE}\left(p,y\right)=\left\{\begin{array}{cc} -\log\left(p\right) & \mathrm{if}\;y=1\\ -\log\left(1-p\right) & \mathrm{otherwise} \end{array}\right.$$

where y=1 or 0, and p∈[0,1] represents the probability that the prediction is positive (y=1). To unify *p* and 1−p, the function $p_{t}$ is defined as,

(3) $$p_{t}=\left\{\begin{array}{cc} p & \mathrm{if}\;y=1\\ 1-p & \mathrm{othe}\mathrm{rwise} \end{array}\right.$$

Therefore, $\mathrm{CE}\left(p,y\right)=\mathrm{CE}\left(p_{t}\right)=-\log\left(p_{t}\right)$.

To address the 0 − 1 imbalance issue in chromatin accessibility data, Focal loss was used.^[^ ^21^ ^]^

(4) $$\mathrm{FL}\left(p_{t}\right)\;=-\alpha_{t}{\left(1-p_{t}\right)}^{\gamma }\log p_{t}$$

where ${\left(1-p_{t}\right)}^{\gamma }$ is a modulating factor with a tunable focusing parameter γ≥0; and the weighting factor α∈[0,1] is for class 1 and 1−α is for class 0. The focusing parameter γ balanced the hard and easy samples, while the weighting factor α balanced the ratio of 0 − 1 samples. The relationship between the different parameter settings and the magnitude of the loss function was manually examined for the first few training epochs to determine the optimal values. Based on this examination, α=0.93 and γ=2 were set for all the results described in the study.

The RNA decoder produced the mean (μ) and dispersion (θ) parameters of the negative binomial components for each gene. The NB distribution parameterized the likelihood of observing the measured expression *x* through these two parameters.

(5) $$\mathrm{NB}\left(x;\mu ,\theta \right)\;=\frac{\tau \left(x+\theta \right)}{\tau \left(\theta \right)}\;{\left(\frac{\theta }{\theta \;+\;\mu }\right)}^{\theta }{\left(\frac{\mu }{\theta \;+\;\mu }\right)}^{x}$$

where τ denotes the gamma function.

To maximize the likelihood of observed data, the following loss function was employed.

(6) $$\begin{array}{ccc} \mathrm{NBloss}\left(x;\mu ,\theta \right)\; & = & \;-\theta \left(\log\left(\theta +\varepsilon \right)-\log\left(\theta +\mu \right)\right)\\ & & -x\left(\log\left(\mu +\varepsilon \right)-\log\left(\theta +\mu \right)\right)\\ & & -\log\tau \left(x+\theta \right)+\log\tau \left(x+1\right)+\log\tau \left(\theta +\varepsilon \right) \end{array}$$

where ε is a small constant of numerical stability.

In the pre‐training phase, the RMSProp optimization algorithm was used, while in the training phase, the model was trained using the Adam optimizer with a batch size of 256 and a learning rate of 0.01. Early stopping was performed based on the loss variation in the validation set.

### Benchmark Evaluation Metrics

In order to assess the quality of generated ATAC data, AUROC was used since chromatin accessibility data were binarized. AUROC was a value between 0 and 1, where a higher value indicated better classification performance for positive and negative samples. Conversely, RNA expression was considered continuous, and therefore, the quality of generated RNA expression was evaluated using Pearson's correlation (on log‐scaled expression), which measured the linear correlation between two variables *X* and *Y* and ranges from −1 to 1, with a higher value indicating a stronger correlation. All metrics were calculated using the Python library scikit‐learn.^[^ ^35^ ^]^

### Unpaired Data Analysis

In the process of generating RNA from ATAC, LiftOver^[^ ^36^ ^]^ was used to convert the coordinates Hg19 to Hg38 before feeding the data into scMOG. The Signac R package^[^ ^37^ ^]^ was employed for cell type annotation in ATAC data, while the Scanpy Python package^[^ ^32^ ^]^ was utilized for RNA data preprocessing, Leiden downscaling, and UMAP visualization.

On the other hand, for generating ATAC from RNA, Scanpy was used for RNA data preprocessing to obtain the cell type annotations. To ensure the quality of the generated ATAC data, Signac was applied to remove peaks that were expressed in less than 10 cells and cells with less than 200 accessible peaks. In addition, latent semantic indexing (LSI)^[^ ^38^ ^]^ was utilized for dimensionality reduction before applying UMAP visualization.

### Joint Analysis of Multi‐Omics Data

Seurat was utilized to conduct a joint analysis of both the generated ATAC data and the measured RNA data. To annotate cell types within the dataset, the tools available in the Seurat were utilized, specifically transferring cell labels from the existing PBMC reference dataset.^[^ ^39^ ^]^ The CoveragePlot() function was then employed to visually represent both gene expression and chromatin accessibility data, and FindMotifs() was used to identify motifs specific to different cell types. To identify differentially accessible regions among clusters of cells, a differential accessibility test was performed,^[^ ^40^ ^]^ and these regions were subsequently represented on the UMAP plots.

### Expansion to Proteomics

Table S5, Supporting Information summarizes the two datasets used in this study, comprising 7865 cells and 5527 cells, respectively. Both datasets consist of paired scRNA‐seq and surface protein counts obtained from human PBMC and measure 33 538 genes and 17 proteins. The same preprocessing pipeline was employed for the RNA data as described in the “Datasets and Pre‐Processing” section. Concerning the surface protein counts, the relative abundance transformation method was utilized,^[^ ^8^ ^]^ which performed a centered log‐ratio transformation on the protein counts of each cell. Specifically, for each cell *c*, the vector *z_c_* was computed as follows.

(7) $$z_{c}=\;\left[\ln\left(\frac{t_{1c}}{g\left(t_{c}\right)}\right),\;\ln\left(\frac{t_{2c}}{g\left(t_{c}\right)}\right)\text{…}\;\ln\left(\frac{t_{\mathrm{dc}}}{g\left(t_{c}\right)}\right)\right]$$

where *t*_c_ is a vector of antibody‐derived tags (ADT) counts, and $g(t_{c})$ is the geometric mean of *t*_c_. It was observed that transforming the protein counts using a centered log‐ratio transformation method enhanced the generative performance of the network in comparison to training the network with the raw protein counts.

To generate proteins, protein decoder networks were utilized, which took a 16D latent representation of the RNA encoder as an input and yielded normalized counts of proteins. The decoder consisted of three fully connected layers, including a 16D input layer, a 64D hidden layer, and a 17D output layer. In each layer of the decoder, except for the last one, the LeakyRelu activation function was used. The final layer was a fully connected layer with an identity activation function for output. In addition, a protein discriminator was built to perform pre‐training. The discriminator comprised fully connected layers, mapping from a 17D input layer and two hidden layers with 256 and 64 dimensions to a 1D output layer. During the training phase, the loss function was set to mean square error loss. For the generated protein data, the Scanpy package was used for clustering visualization, violin plot, and heatmap plotting.

## Conflict of Interest

The authors declare no conflict of interest.

## Supporting information

Supporting Information

## Data Availability

The data that support the findings of this study are available in the supplementary material of this article. All Python and R codes used in this study are publicly available at https://github.com/GaoLabXDU/scMOG.
