# Supplementary material for: Efficient Generation of Paired Single‐Cell Multiomics Profiles by Deep Learning
Source: Adv Sci (Weinh). 2023 Apr 28;10(21):2301169. doi: 10.1002/advs.202301169 (PMC10375161; doi:10.1002/advs.202301169)
Supplement: Supplementary file 1 — Supporting Information [file ADVS-10-2301169-s001.pdf]

ADVANCED SCIENCE
Open Access

Supporting Information

for *Adv. Sci.*, DOI 10.1002/advs.202301169

Efficient Generation of Paired Single-Cell Multiomics Profiles by Deep Learning

Meng Lan, Shixiong Zhang and Lin Gao**

Supplementary of "Efficient generation of paired single-cell multiomics profiles by deep learning"

Table S1. Summary of the datasets for single-cell transcriptome and chromatin accessibility cross generation.

	Cell population	Technology & GEO accession number (if applicable)	Cells	Genes	Peaks
Paired datasets	Human PBMC	10X	11,909	36,601	108,377
	Human DM, HSR	GSE160148	24,508	32,739	363,027
	Mouse brain cerebral cortex	SNARE-seq GSE126074	10,309	31,499	239,241
	Mouse brain	SHARE-seq GSE140203	3,293	21,127	428,041
	Human PBMC	10X	10,691	36,601	115,179
	Flash-Frozen Lymph Node with B Cell Lymphoma	10X	14,566	36,601	109,789
	Alzheimer's Disease Mouse Brain	10X	33,459	32,286	66,914
	Mouse brain nuclei	10X	23,990	32,285	80,935
	GM12878	GSE166797	3,509	36,601	129,346
Unpaired datasets	Human PBMC	10X	8,633		80,234
	Human PBMC	10X	4,623		135,377
	Mouse brain nuclei	10X	7,377	32,285	
	Human PBMC	10X	5,140	36,601	

Table S2. scMOG generation performance across different held-out test clusters. Randomly divide the human PBMC (11,909 cells) and GSE160148 (24,508 cells) datasets into five folds for cross-validation. We observe consistent performance across all folds.

	**Metric**	Fold 1	Fold 2	Fold 3	Fold 4	Fold 5
**ATAC > RNA**	Pearson's r	0.7321	0.7349	0.7319	0.7301	0.7311
**RNA > ATAC**	AUROC	0.9059	0.9061	0.9038	0.9053	0.9065

Table S3. Evaluation of the effectiveness of the number of peak calling features on RNA generation performance using scMOG on the SHARE-seq mouse brain dataset (consisting of 3,293 cells and 200,255 peaks after preprocessing) by measuring the Pearson's correlation coefficient (Pearson's r) between generated RNA and measured RNA in both the original and downsampled dimensions.

**ATAC -> RNA**	Peaking calling features	200,255	100,000	50,000	10,000	5,000
	Pearson's r	0.7049	0.7022	0.7007	0.6918	0.6527

Table S4. Benchmark evaluation of scMOG, using multiple datasets from mouse and human in both within dataset and across dataset scenarios, compared to No_Pretrain model.

	**Model**	**Training scheme**		**ATAC>RNA**: Pearson's correlation	**RNA>ATAC**: AUROC
		Cell population	Technology		
**Within dataset**	scMOG validation	PBMC, DM, HSR	10X	0.84	0.88
	No_Pretrain validation			0.82	0.87
	scMOG validation	Mouse Brain	SNARE-seq	0.57	0.81
	No_Pretrain validation			0.56	0.79
**Across dataset**	scMOG	Lymphoblastoid		0.62	0.80
	No_Pretrain_model			0.59	0.72
	scMOG	PBMC	10X	0.57	0.89
	No_Pretrain_model			0.56	0.84

Table S5. Summary of the datasets for cell surface protein generation from RNA.

	**Cell population**	**Technology**	**Cells**	**Genes**	**Proteins**
**Paired datasets**	Human PBMC	10X Chromium Demonstration (v2 Chemistry)	7,865	33,538	17
	Human PBMC	10X Chromium Next GEM Demonstration (v3.1 Chemistry)	5,527	33,538	17

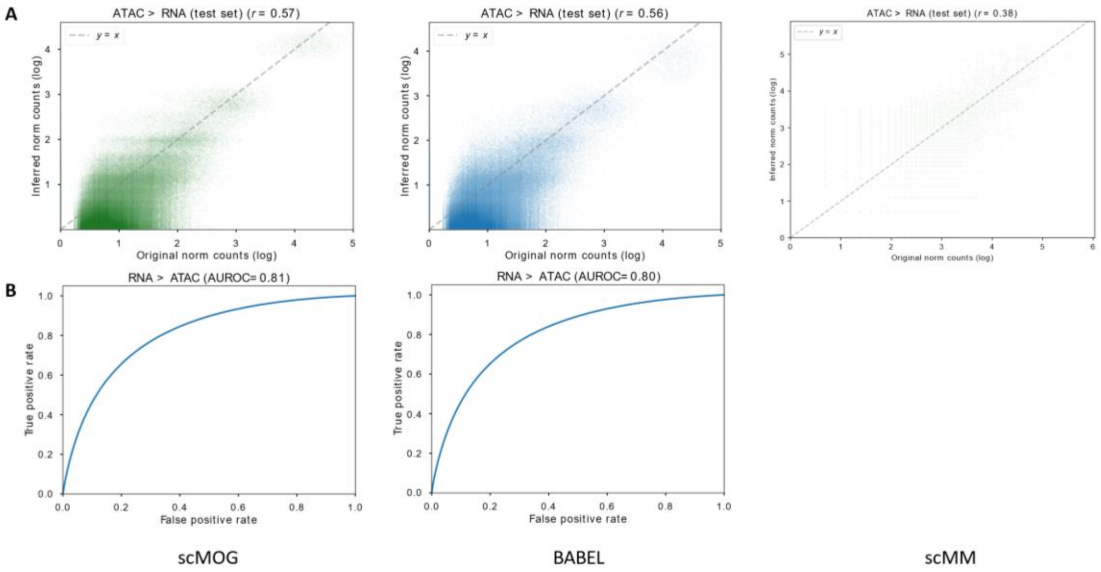

Figure S1. scMOG's performance when trained and tested on SNARE-seq mouse data (10,309 cells) and compared to BABEL and scMM. (A) Evaluation of the generation performance of ATAC to RNA using Pearson's correlation. (B) Evaluation of the generation performance of RNA to ATAC using AUROC.

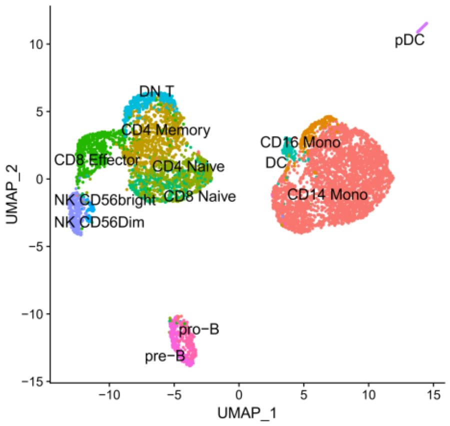

Figure S2. The UMAP plot for scATAC-seq human PBMC data (8,633 cells). The UMAP plot is used as a benchmark to see how these are retained in the generated RNA expressions.

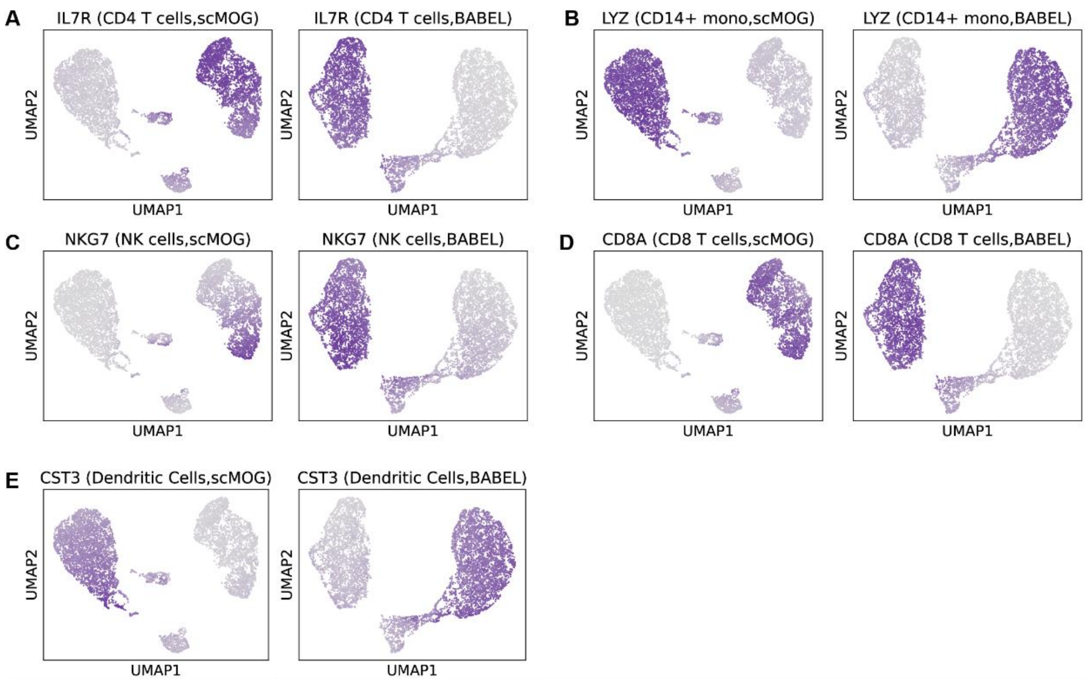

Figure S3. Visualization of the marker gene activity on RNA expression generated by scMOG and BABEL from scATAC-seq human PBMC data (8,633 cells). Feature plots for (A) IL7R (a marker for CD4 T cells), (B) LYZ (a marker for CD14+ monocytes), (C) NKG7 (a marker gene for NK cells), (D) CD8A (a marker for CD8 T cells), and (E) CST3 (a marker for DC cells). The scatter plot is a UMAP representation of the generated RNA expression. We colored each cell according to the gene expression generated by scMOG and BABEL.

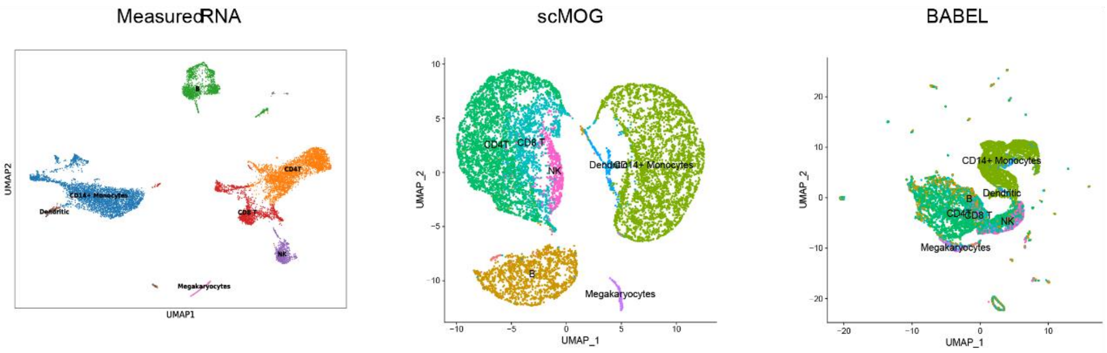

Figure S4. UMAP plots of measured RNA (left), scMOG's generated ATAC (middle) and BABEL's generated ATAC (right) from scRNA-seq human PBMC data (11,769 cells), where the generated ATAC is not processed with quality control. Each cell is colored according to measured RNA expression. The UMAP plot of RNA is used as a benchmark to see how these are retained in the generated ATAC expression.

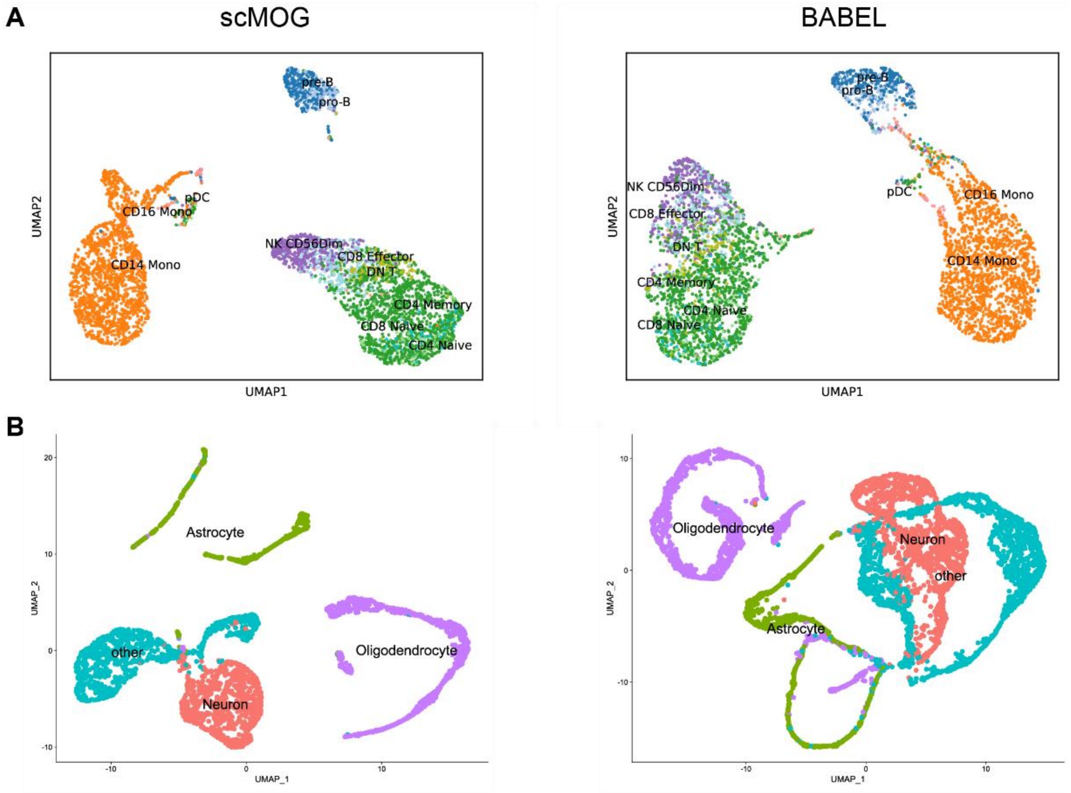

Figure S5. (A) UMAP plots of scMOG's generated RNA (left) and BABEL's generated RNA (right) from scATAC-seq human PBMC data (4,623 cells). Each cell is colored according to measured ATAC. (B) UMAP plots of scMOG's generated ATAC (left), and BABEL's generated ATAC (right) from scRNA-seq mouse brain nuclei data (7,377 cells). Each cell is colored according to measured RNA expression.

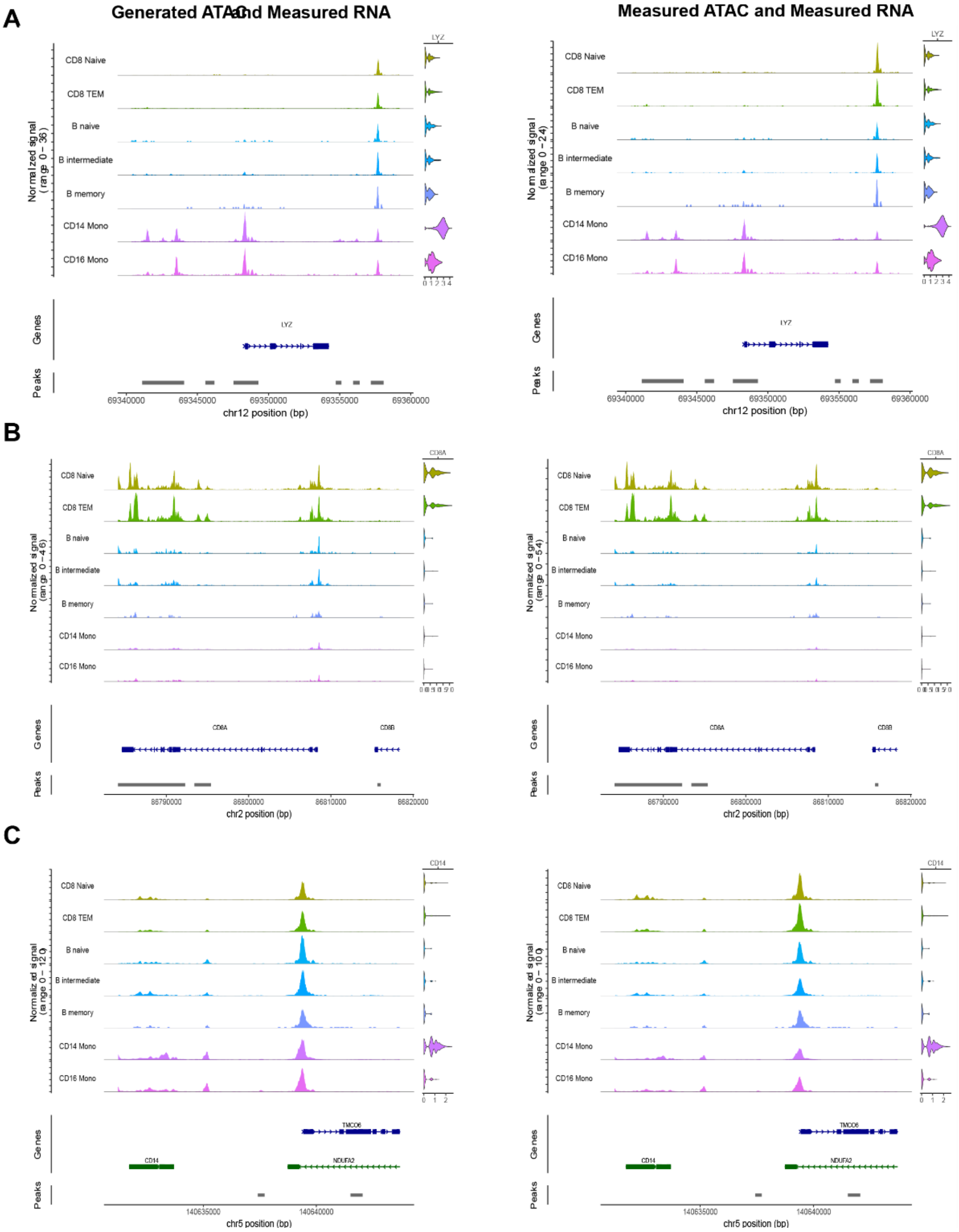

Figure S6. The coverage plots on paired human PBMC data (10,691 cells). The coverage plots of clusters within regions of (A) LYZ (a marker for CD14+ monocytes), (B) CD8A (a marker for CD8 T cells) and (C) CD14 (a marker for CD14+ monocytes) for the generated ATAC and measured RNA (left), and the measured ATAC and measured RNA (right).

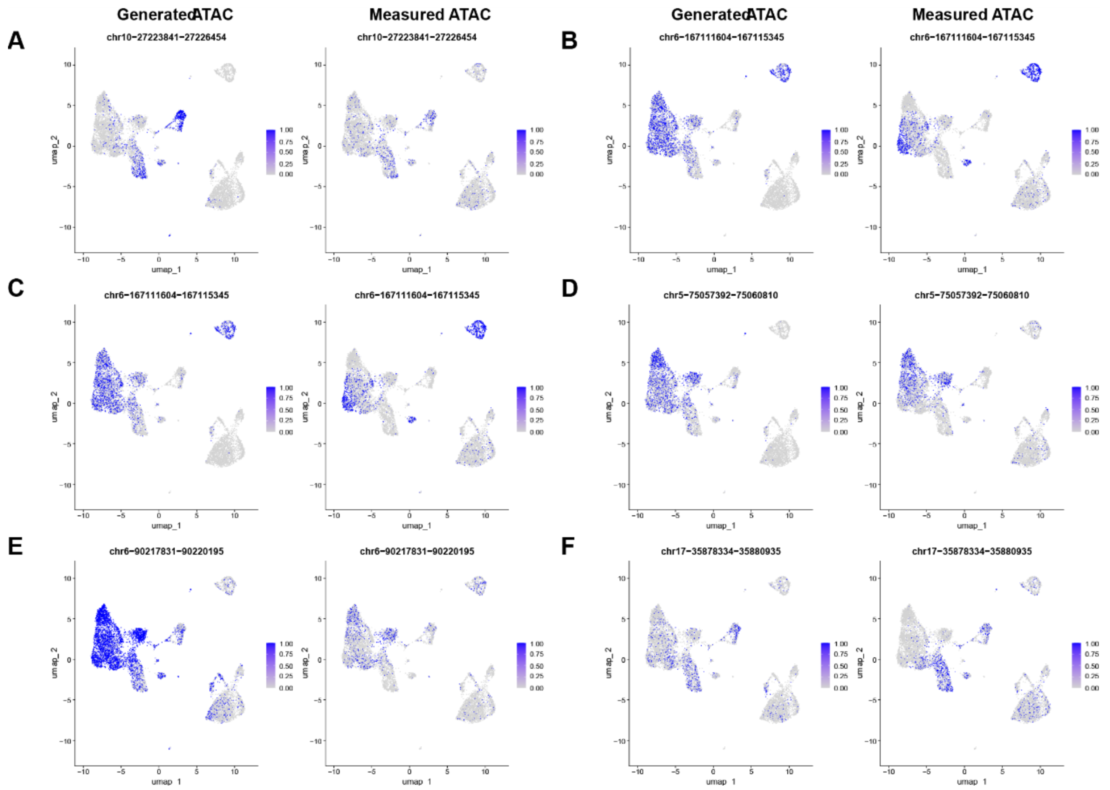

Figure S7. The differentially accessible regions on paired human PBMC data (10,691 cells). (A-F) highlight differentially accessible regions between cell types for generated ATAC (left) and measured ATAC (right). The cell types include (A) CD14 Mono and NK, (B) CD14 Mono and B naive, (C) NK and B naive, (D) CD4 naive and CD4 TEM, (E) CD8 naive and CD16 Mono, (F) CD8 naïve and CD8 TEM.

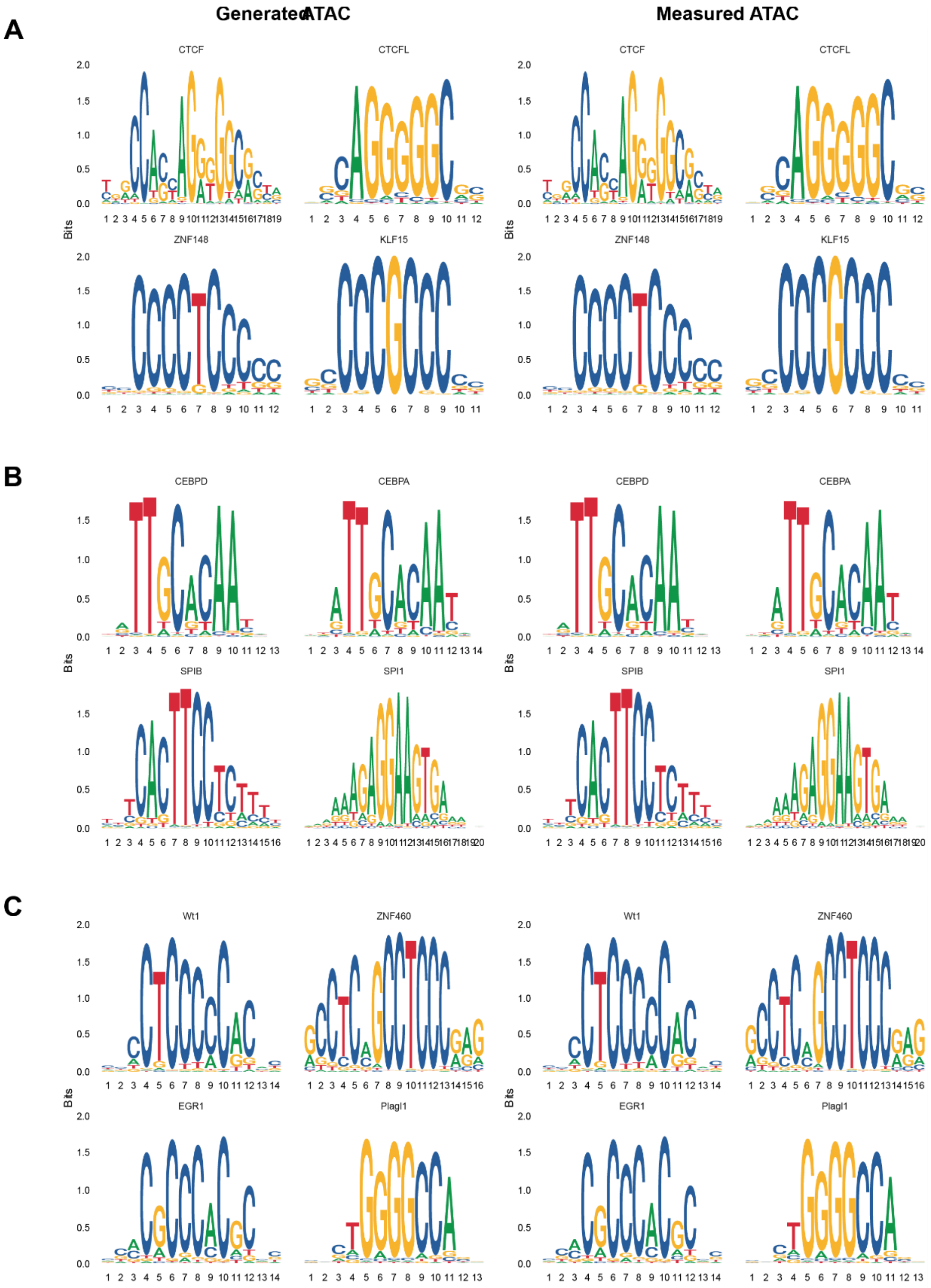

Figure S8. Motif plots for representative motifs from generated ATAC (left) and measured ATAC (right) between (A) NK and B naive, (B) CD14 Mono and B naive, (C) CD4 naive and CD4 TEM.

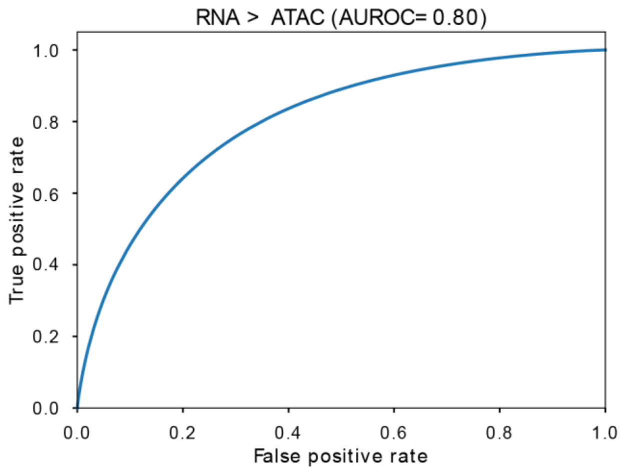

Figure S9. RNA to ATAC generation performance evaluation using AUROC when trained and tested on lymph node tumor data (14,566 cells).

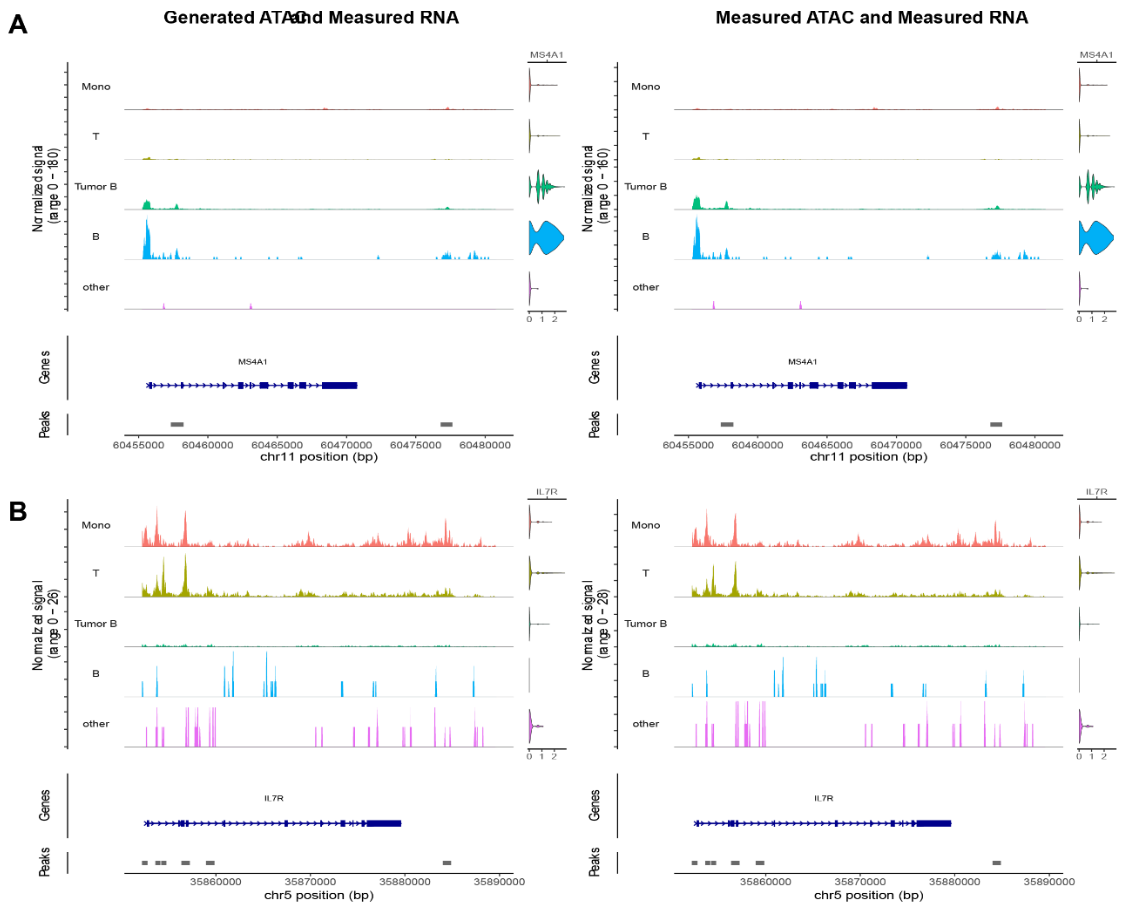

Figure S10. The coverage plots on lymph node tumor data (14,566 cells). The coverage plots of clusters within regions of (A) MS4A1 (a marker for B cells), and (B) IL7R (a marker for CD4 T cells) for the generated ATAC and measured RNA (left), and the measured ATAC and measured RNA (right).

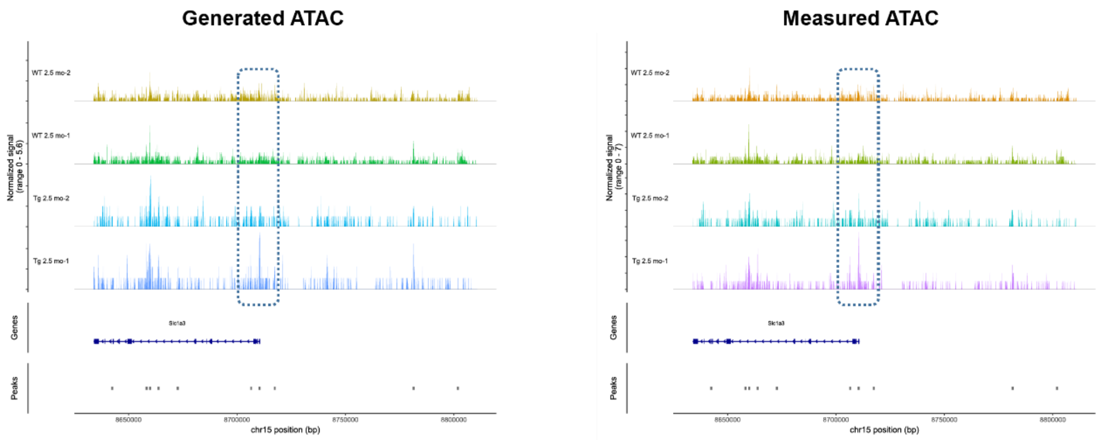

Figure S11. Comparison of the coverage plots of Slc1a3 between the generated ATAC (left) and the measured ATAC (right) on Alzheimer's disease mouse brain dataset, which contains 33,459 cells. The comparison is done for two transgenic mice (Tg 2.5 mo-1 and Tg 2.5 mo-2) and two wild-type mice (WT 2.5 mo-1 and WT 2.5 mo-2) at 2.5 months of age. The coverage plots demonstrate the heterogeneity among individuals, where Slc1a3 exhibits differences between Tg and WT mice. One such difference is marked by a dashed line.

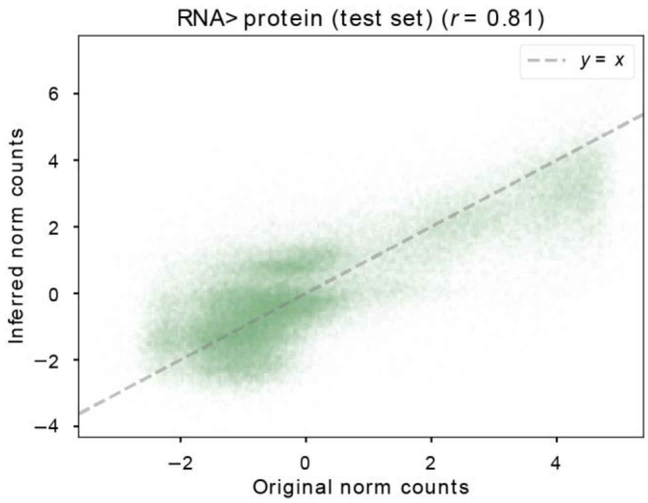

Figure S12. RNA to protein generation performance evaluation using Pearson's correlation when tested on human PBMC datasets (5,527 cells).

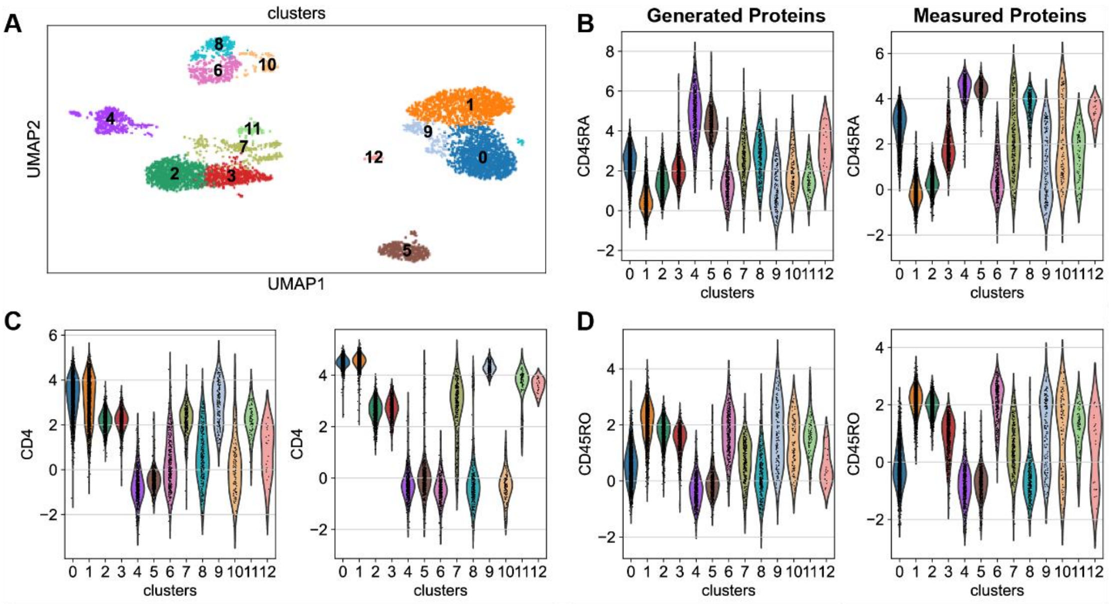

Figure S13. Violin plot of the protein generated by scMOG and the experimentally measured from PBMC human data (5,527 cells). (A) UMAP plots of measured protein. We used the 13 clusters in the UMAP plot as the x-axis of the violin plot for analysis. (B-D) Violin plots visualizing the feature values of (B) CD45RA, (C) CD4, and (D) CD45RO. We examine the generated expression (left) and measured expression (right).
